# Characterization of Varicella-Zoster (VZV) Specific T Cell Response in Healthy Subjects and Transplanted Patients by Using Enzyme Linked Immunospot (ELISpot) Assays

**DOI:** 10.3390/vaccines9080875

**Published:** 2021-08-06

**Authors:** Irene Cassaniti, Alessandro Ferrari, Giuditta Comolli, Antonella Sarasini, Marilena Gregorini, Teresa Rampino, Daniele Lilleri, Fausto Baldanti

**Affiliations:** 1 Molecular Virology Unit, Microbiology and Virology Department, IRCCS Policlinico San Matteo, 27100 Pavia, Italy; i.cassaniti@smatteo.pv.it (I.C.); alessandro.ferrari@smatteo.pv.it (A.F.); g.comolli@smatteo.pv.it (G.C.); a.sarasini@smatteo.pv.it (A.S.); fausto.baldanti@unipv.it (F.B.); 2 Department of Clinical, Surgical, Diagnostic and Pediatric Sciences, University of Pavia, 27100 Pavia, Italy; 3 Laboratory of Biochemistry-Biotechnology and Advanced Diagnostics, Fondazione IRCCS Policlinico San Matteo, 27100 Pavia, Italy; 4 Unit of Nephrology, Dialysis and Transplantation, Fondazione IRCCS Policlinico San Matteo, 27100 Pavia, Italy; m.gregorini@smatteo.pv.it (M.G.); t.rampino@smatteo.pv.it (T.R.); 5 Department of Internal Medicine and Therapeutics, University of Pavia, 27100 Pavia, Italy

**Keywords:** varicella-zoster virus, T effector and T central memory response, transplant

## Abstract

Solid organ transplant recipients, due to the administration of post-transplant immunosuppressive therapies, are at greater risk of viral reactivation episodes, mainly from herpes viruses, including varicella-zoster virus (VZV). The aim of this pilot study was to develop functional immunological assays (VZV-ELISpot) for the quantification and characterization of the VZV-specific effector-memory and central-memory responses in healthy subjects and transplanted patients. Glycoprotein gE and immediate-early 63 (IE-63) were used as antigens for in vitro stimulation. VZV-seropositive healthy subjects showed higher responses in respect to seronegative subjects. Even if differences were observed between VZV-seropositive healthy subjects and transplanted subjects at pre-transplant, the VZV-specific T-cell response was reduced at 60 days after transplant, mainly for the high level of immunosuppression. Phenotypical characterization revealed that response against VZV was mainly mediated by CD4 T cells. The results obtained in this study might be useful for the definition of personalized follow-up of the transplanted patients, providing useful information on the status of the patient potentially at risk of viral reactivation or other opportunistic infections.

## 1. Introduction

Varicella-zoster virus (VZV) belongs to the α-herpesvirus family, the causative agent of Chickenpox (a highly contagious exanthematic disease that occurs mainly at a pediatric age and that develops because of primary infection) and shingles, also called zoster, caused by the reactivation of the virus that remains latent in the dorsal sensory ganglia. Knowing the condition of a patient’s immune system is fundamental to understand the risk of severe viral infections and their progression. The risk of VZV reactivation or impaired VZV control, as observed with other herpesviruses, can be detected, especially in patients with immune system deficiencies. Rondaan et al. demonstrated a decreased cellular immunity to VZV in systemic lupus erythematosus patients [1], while other researchers reported similar findings for other patient groups at risk for herpes zoster, including transplant recipients, diabetes mellitus patients and HIV-infected patients [2,3,4]. Complications in immunosuppressed subjects are much more frequent and include encephalomyelitis, cerebellar ataxia, arthritis, hepatitis, hemorrhagic nephritis, myocarditis, otitis media [5,6,7,8,9,10,11]. It has been suggested that low antibody titers or B cell immunodeficiency are not related to severe chickenpox cases [12]. On the other side, the development of VZV-specific T cells seems to be necessary to prevent disseminated infection and resolution of acute infection, as suggested by the risk of potentially lethal varicella in children with neoplasms, congenital T-cells immunodeficiency, or with immunosuppressive treatment following organ transplantation. 

In our pilot study, we characterized two immunological approaches for the quantification of VZV-specific T-cell response. Specifically, two methods for the quantification of VZV-specific T cells, known as ex vivo or standard ELISpot assay and cultured ELISpot assay, were designed. Overall, they allow, with a good estimation, the identification of effector memory (Tem) and central memory (Tcm) T-cells, respectively. In detail, ex vivo ELIspot assay allows the identification of cells with immediate secretion functions that are thought to be mainly Tem cells [13]. From a functional point of view, Tem are able to rapidly migrate to the infected peripheral tissues, where they perform their effector action [14]. On the other side, cultured ELISpot assay is based on a previous in vitro lymphocyte culture followed by ELISpot assay, thus allowing a long-period stimulation of T cells. In this setting, the assay may allow the identification of Tcm that have little or no effector function but, conversely, in the presence of their antigen, proliferate rapidly and differentiate into effector T cells. Other than the functional differences between the two memory cell pools, phenotypical differences have also been described [15]. Tcm cells express the CCR7 and CD62L receptors required for lymph nodes migration, which are absent on Tem cells. Tem have poor proliferative capacity, but strong secretory properties of effector molecules, such as IFN-γ and cytolytic molecules, such as perforin; otherwise, Tcm do not immediately produce high levels of effector molecules, but only because of secondary stimulation, but proliferate extremely rapidly [14]. 

In a clinical setting, over 90% of adult solid organ transplant (SOT) recipients are seropositive for varicella-zoster virus (VZV). This means that almost the entire adult population has experienced a primary VZV infection. Rates are lower in pediatric transplants [16,17]. Our study improves the knowledge in terms of cell-mediated response, opens new issues for the study and characterization of antigen-specific T lymphocyte activity by evaluating the secretory activity of certain cytokines, which is useful for understanding the individual ability to control infection and defines the risk of viral reactivation. Given the preventive role of systemic VZV-specific T-cell immunity, the characterization of the circulating VZV-reactive T-cell subsets is of potential diagnostic value [18]. Overall, the aim of this study was to design an immunological assay for the characterization of VZV-specific T-cell response in healthy subjects and transplanted patients.

## 2. Materials and Methods

### 2.1. Subjects Enrolled

Residual stored samples obtained from 35 healthy VZV-seropositive and 7 VZV-seronegative healthy subjects (median age 36, range 25–48 years; 14 males and 21 females and median age 28 ± 3 years; 6 males and 1 female, respectively) were used for the setting of immunological assays. Twelve VZV-seropositive kidney transplant recipients (KTR; median age 58, range 48–85; 10 males and 2 females) enrolled at the time of transplant and after 60 and 180 days were also considered in the study.

The study was performed according to the guidelines of the Declaration of Helsinki and approved by the Ethics Committee of Fondazione IRCCS Policlinico San Matteo (proc no. 38433/2017).

### 2.2. Isolation of Peripheral Blood Mononuclear Cells

Peripheral blood mononuclear cells (PBMCs) were isolated by standard density gradient centrifugation using Lymphoprep (Lymphoprep, Axis-Shield, Oslo, Norway) from heparinized whole blood samples. Isolated PBMCs were then cryopreserved in fetal calf serum (FCS; Euroclone, Milan, Italy) supplemented with 10% dimethyl sulfoxide (Sigma-Aldrich, Darmstadt, Germany) and preserved in liquid nitrogen. After thawing, viable cells were counted and used in ex vivo and/or cultured ELISpot and intracellular cytokine staining (ICS) assay.

### 2.3. Antibody Titer

For quantification of anti-VZV antibodies in plasma, the automated chemiluminescent microparticle immunoassay system (CMIA, Architect system, Abbott Laboratories, IL, USA) was used. A positive result for VZV IgG was considered when in the presence of at least 135 U/mL.

### 2.4. Synthetic Peptides and ELISpot Assay

For the ELISpot set-up, two pools of lyophilized synthetic peptides (15 mers with 11 amino acids overlap) were used, spanning the glycoprotein E (gE, 153 peptides) and the Immediate-early protein 63 (IE63; 67 peptides) (JPT, innovative peptide solution, Berlin, Germany). Each peptide pool was dissolved in Dimethyl sulfoxide (DMSO) and diluted in an RPMI medium. In each experiment, a final concentration of 0.25 μg/mL of each peptide was used. Human interferon-gamma (IFN-y) ELISpot kits (Diaclone, Cedex, France) and MultiScreen-IP membrane-bottomed 96-well plates (Merck Millipore, Darmstadt, Germany) were used for standard ELISpot assays and for cultured ELIspot assays according to the previous protocols, with some modifications [13,19].

For ex vivo ELISpot assays, the plates were coated overnight with a monoclonal capture antibody against IFNγ and stored at 4 °C. After washing with PBS, the plates were blocked with culture medium (RPMI 1640 supplemented with 2 mM L-glutamine, 100 U/mL penicillin and 100 μg/mL streptomycin, and 10% heat-inactivated fetal bovine serum (FBS) (Euroclone, Milan, Italy)) for 2 h at room temperature. Cells were plated in duplicate (/100 μL per well) and stimulated with the corresponding antigens or with phytohemagglutinin (PHA, 5 μg/mL, Sigma-Aldrich) or with medium alone (negative control) and incubated at 37 °C in a 5% CO_2_ humidified atmosphere for 24 h. After washing, the plates were incubated at 37 °C for 90 min with a biotinylated IFN-γ detection antibody. The plates were then washed, and streptavidin-alkaline phosphatase conjugate was added. The plates were incubated at 37 °C in a 5% CO_2_ atmosphere for 60 min. After washing, 5-bromo-4-chloro-3-indolyl phosphate/nitro blue tetrazolium (BCIP/NBT) was added for 20 min at room temperature. The wells were then washed several times under running water and air-dried overnight. Spots were counted by using an automated AID ELISPOT reader system (Autoimmun Diagnostika GmbH, Strasburg, Germany). The mean number of spots from duplicate were adjusted to 1 × 10^6^ PBMC. The net spots per million PBMC was calculated by subtracting the number of spots in response to negative control from the number of spots in response to the corresponding antigen.

For the cultured ELISpot assays, PBMC were resuspended in a cultured medium and rested overnight at 37 °C in a 5% CO_2_ humidified atmosphere. Then, PBMC were cultured (1 × 10^6^ cells/mL per well) in 24-well plates (Becton Dickinson, NJ, USA) and stimulated with gE or IE63 pools or medium alone. The plates were incubated for 10 days. On days 3 and 7, 500 µL of the medium was removed and replaced with 500 µL of cultured medium containing 20 IU/mL of recombinant human interleukin-2 (Peprotech, London, UK). On day 11, cells from each well were washed, resuspended in the culture medium at a concentration of 1 × 10^6^/mL and kept overnight at 37 °C humidified atmosphere 5% CO_2_ before the ELISpot procedure, as previously described.

### 2.5. Intracellular Cytokine Staining (ICS)

The available number of PBMC allowed further analysis of eight samples by flow cytometry. In detail, 0.5 × 10^6^ cells were transferred to a 96-well round-bottom plate in 100 µL culture medium with fetal calf serum in the presence of the corresponding peptide pool or SEB or culture medium alone. Following 1-h incubation at 37 °C in a humidified 5% CO2, brefeldin A (Sigma-Aldrich) at a final concentration of 10 ug/mL was added. After overnight incubation, cells were washed in phosphate-buffered saline (PBS) ethylenediaminetetraacetic acid (EDTA) 2mM and incubated with the Live/Dead Fixable Far Red Dead Cell Stain Kit (ThermoFisher Scientific, Waltham, MA, USA) for 30 min at 4 °C. Cells were then washed with PBS, fixed and permeabilized with BD Cytofix/Cytoperm (BDBiosciences, San Jose, CA, USA) according to the manufacturer’s instructions for intracellular staining with the following monoclonal antibodies: IFNγ-FITC, CD4-ECD, CD8-PC7, CD3-Pe-Cy5 (all from BeckmanCoulter, Brea, CA, USA). Finally, the cells were resuspended in 4% paraformaldehyde and analyzed with a Navios flow cytometer (Beckman Coulter, Brea, CA, USA), obtaining the percentages of IFN-γ secreting lymphocyte cell responses and allowing phenotypical discrimination of individual cytokine-producing cells

### 2.6. Data Analysis

For each condition tested in duplicate, the mean of spots obtained was calculated, adjusting the number to 1 million PBMC. Net spots/million PBMC was obtained by subtracting the number of spots in wells with culture medium only from the number of spots in wells from each VZV peptide pool. The proliferation index (PI) for each tested condition was also calculated in cultured ELISpot assay, as the ratio of the number of cells proliferated during the 12 days of culture stimulated with PHA or gE or IE63 and the number of cells proliferated during the same period in the presence of the culture medium only. All results were shown in terms of net spots/million PBMC multiplied by the PI (net spots /million PBMC×PI). For the comparison of two groups, quantitative variables were analyzed using the Mann–Whitney U test. The analysis of the variations with time of antigen-specific response in transplant recipients was performed with the Friedman test for repeated measures. Finally, the correlation between T-cell responses and the antibody titer was performed using Spearman tests, as well as the correlation between responses measured by ex vivo and cultured ELISpot against gE and IE63 specific T-cell response. All analyses were performed using GraphPad Prism software (version 5; GraphPad Software Inc., La Jolla, CA, USA). Results with *p* < 0.05 were considered statistically significant.

## 3. Results

### 3.1. gE and IE63-Specific T-Cell Response Measure by Ex Vivo ELISpot Assay in Healthy Subjects

**Figure 1:**
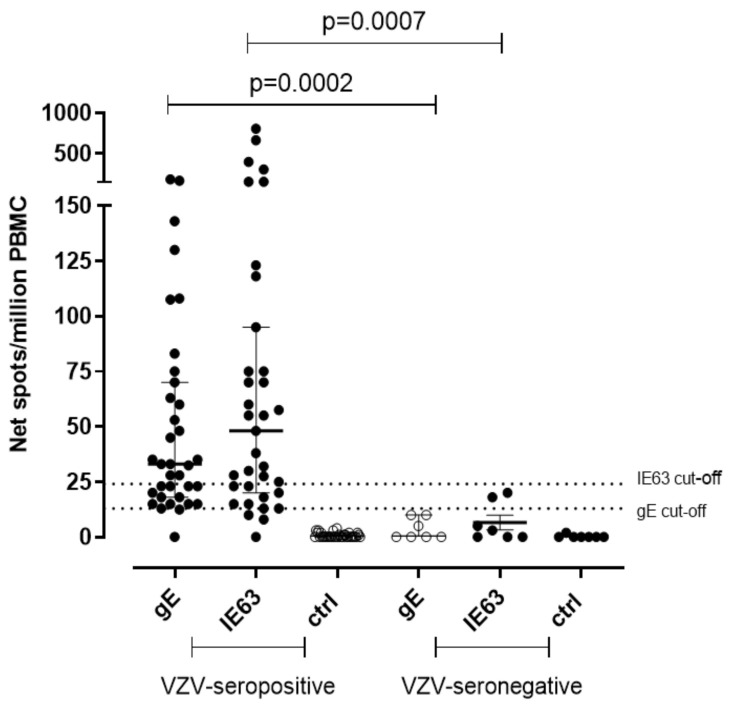
Ex vivo ELISpot assay for VZV-specific T-cell response. VZV-specific T-cell response to gE and IE63 35 VZV-seropositive healthy subjects and 7 VZV-seronegative healthy subjects. The cut-off of positive responses was calculated as the mean of the response in VZV-seronegative subjects plus two standard deviations. Ctrl: Ex vivo ELISpot assay measured against medium only.

A significantly higher T-cell response specific for gE and IE63 was observed in seropositive subjects. In detail, median gE-specific T-cell response was 33 (IQR 18–70) net spot/million PBMC in seropositive subjects (*p* = 0.0002) and was 0.0 (IQR 0–10) net spot/million PBMC in seronegative controls. IE63-specific T-cell response was 48 (IQR 20–95) and 3 (IQR 0–18) net spot/million PBMC in seropositive and seronegative, respectively (*p* = 0.007) (Figure 1). The cut-off for positive responses was calculated on the mean of gE and IE63-specific T-cell response of seronegative controls plus two standard deviations (SD) (13 and 24 net spot/million PBMC, respectively).

### 3.2. gE and IE63-Specific T-Cell Response Measure by Cultured ELISpot Assayin Healthy Subjects

**Figure 2:**
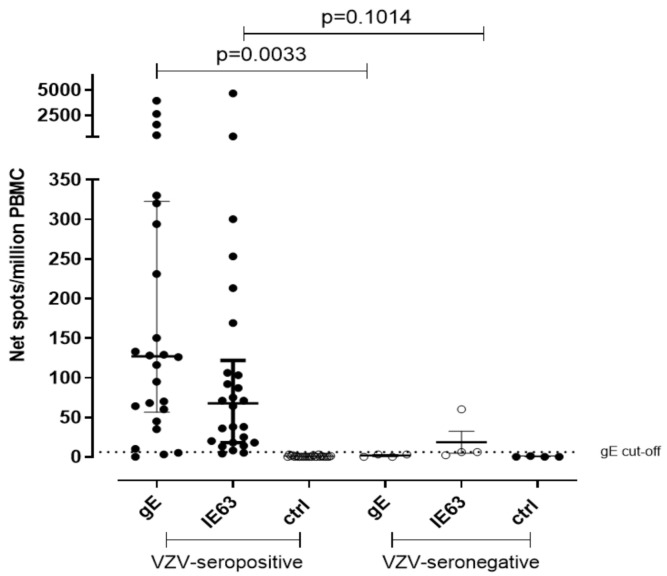
Cultured ELISpot for VZV-specific T-cell response. VZV-specific T central memory response to gE and IE63 in 26 VZV-seropositive healthy subjects and 4 VZV-seronegative healthy subjects. Ctrl: Ex vivo ELISpot assay measured against medium only.

Due to the lack of samples, the T-cell response against VZV gE and IE63 peptide pool was measured in 26 VZV-seropositive healthy volunteers and in 4 VZV-seronegative controls by using cultured ELISpot assay. Median gE-specific T cell response was 127 net spot/million PBMC and 3 net spot/million PBMC, respectively (*p* = 0.0033), while median IE63-specific T cell response was 67.5 and 6 net spot/million PBMC, respectively (*p* = 0.1014) (Figure 2). The cut-off of positive response was calculated from the mean of gE T-cell response of seronegative controls plus two standard deviations (SD) (5 net spot/million PBMC). IE63 T-cell response cut-off was not possible to calculate since only three VZV-seronegative subjects were tested for IE63 due to the lack of cells. The collected data by both ex vivo and cultured ELISpot assays suggest a statistical relevance of gE instead of IE63.

### 3.3. Correlation of Humoral and T-Effector Memory and T-Central Memory Response against VZV in VZV-Seropositive Healthy Subjects

No correlation was observed between IgG VZV-specific antibody titer and gE specific T-cell response measured by ex vivo ELISpot (r^2^ = 0.01; *p* = 0.6880), as well as between VZV-specific IgG antibody titer and IE63-specific T-cell response measured by ex vivo ELISpot (r2 = 0.03; *p* = 0.3911) in the 26 subjects tested (data not shown). Similarity no correlation was observed between VZV-specific IgG antibody titer and gE specific T cell response (r^2^ = 0.02; *p* = 0.0950), as well as between VZV-specific IgG antibody titer and IE63-specific T cell response (r^2^ = 0.079; *p* = 0.2424) when measured by cultured ELISpot assay (data not shown). Moreover, no correlation was observed between the two assays when gE specific T-cell response (r^2^ = 0.079; *p* = 0.2062) and IE-63 T-cell response (r^2^ = 0.025; *p* = 0.4815) were measured (data not shown).

### 3.4. Phenotypical Characterization of VZV-Specific T-Cell Response Measured by Ex Vivo ELISpot Assay in Healthy Subjects

**Figure 3:**
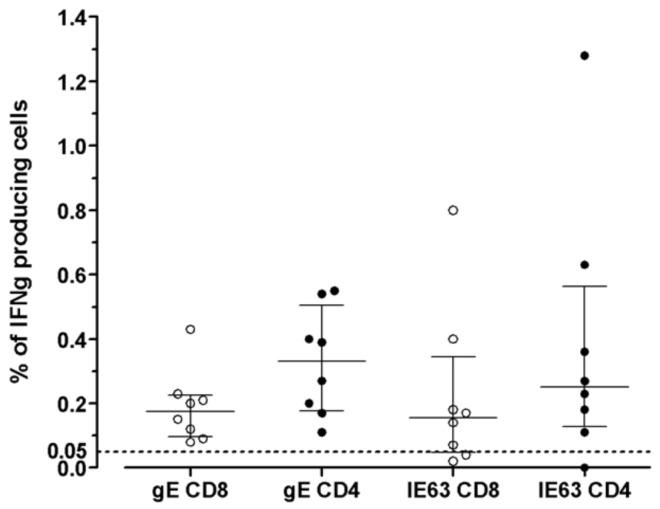
VZV-specific T-cell response to gE and IE63 in 8 VZV-seropositive healthy subjects measured by ICS assay. Results are shown as the percentage of production of IFNγ (cut-off 0.05%).

**Figure 4:**
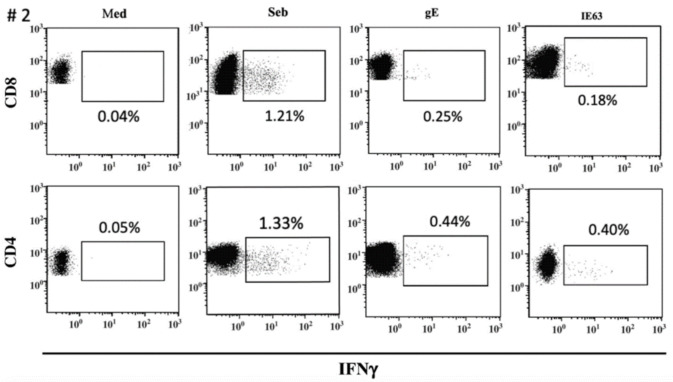
ICS assay VZV-specific T-cell response to gE and IE63 in VZV-seropositive healthy subject #2. Staphylococcus aureus enterotoxin B stimulation (Seb) was used as a positive control.

The distribution of VZV-specific T cells among CD4^+^ or CD8^+^ T-cells subsets was investigated in seven VZV-seropositive by ICS for IFNγ after overnight stimulation with cognate antigens. The median percentage of CD8^+^ T-cells specific for gE antigen was 0.175 (IQR 0.097–0.225), while the median percentage of CD4^+^ T-cells specific for gE antigen was 0.330 (IQR 0.177–0.505). The median percentage of CD8^+^ T-cells specific for IE63 antigen was 0.155 (IQR 0.047–0.345), while the median percentage of CD4^+^ T-cells specific for IE63 antigen was 0.250 (IQR 0.127–0.562). (Figure 3). An example of ICS analysis obtained from VZV-seropositive healthy subjects is shown in Figure 4. As a control, one VZV-seronegative subject was tested by ICS for IFNγ production, and, as expected, no response was observed.

### 3.5. Evaluation of VZV-Specific T-Cell Response in Transplanted Subjects by Using Both Ex Vivo and Cultured ELISpot Assays

**Figure 5:**
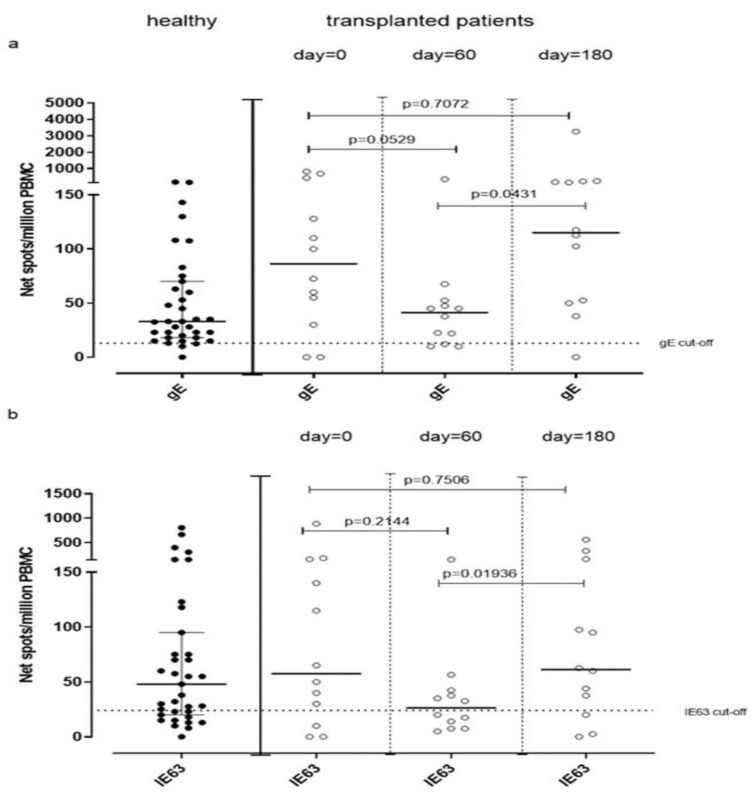
gE (**a**) and IE63 (**b**)-specific T-cell response measured by ex Vivo ELISpot assay have been analyzed before transplant (day 0) and 60 and 180 days post-transplant.

VZV-specific T-cell response measured by ex vivo ELISpot assay was analyzed in 12 VZV-seropositive patients waiting for kidney transplantation (day 0) and compared to VZV-specific T-cell response observed in VZV-seropositive healthy volunteers. The median gE-specific T-cell response was 86 (IQR 36–363) net spot/million PBMC, and median IE63-specific T-cell response was 57 (IQR 15–155) net spot/million PBMC. While median IE63-specific T-cell response was not statistically different from that observed in healthy subjects (*p* = 0.6693), a trend of significance was observed in terms of median gE-specific T-cell response (*p* = 0.0569). VZV-specific T-cell response measured by cultured ELISpot assay in these patients was not evaluated due to the small amount of PBMC obtained from patients. Twelve kidney transplant recipients were monitored during the first six months post-transplant for VZV-specific T-cell response at 60 and 180 days post-transplant. A decrease of gE-specific T-cell response was observed at day 60 (41 (IQR 11.25–51.25) net spot/million PBMC) (*p* = 0.0529), which increased significantly at day 180 (115 (IQR 31.88–181.90) net spot/million PBMC; *p* = 0.0431) (Figure 5a). Similarly, IE63-specific T-cell response significantly changed between day 60 and 180 since it was 34 (IQR 9.37–50.38) net spot/million PBMC and 60 (IQR 17–96.88) net spot/million PBMC (*p* = 0.0194), respectively (Figure 5b).

## 4. Discussion

This report describes the set-up of an in vitro immunological method for the quantification of VZV-specific T-cell response, using two approaches defined as ex vivo and cultured ELISpot assay that are thought to stimulate mainly T effector memory and T central memory responses, respectively [13]. The first result obtained in our study confirms that VZV-seropositive healthy subjects showed a sustained T-cell response against gE and IE63 antigens by using both ELISpot approaches. On the other side, an undetectable response was reported in VZV-seronegative patients indicating the existence of a pool of T memory cells developed in response to the primary infection [20,21]. Even if the large majority of the seropositive subjects showed positive responses to both antigens, two of them showed a negative ex vivo ELISpot response against gE peptide pool, also in the presence of a high level of antibodies; however, a sustained response was reported in these two cases when VZV-specific response was measured by using cultured ELISpot assay. On the other side, two seropositive subjects showed positive VZV-response to gE peptide pool in ex vivo ELISpot assay but not when the response was detected by cultured ELISpot assay, thus supporting the concept that these two assays detected different memory cell subsets. The collected data obtained from both immunological approaches suggest that gE elicits a higher T-cell response than IE63. However, it has to be considered that we analyzed the T-cell response only against two selected proteins and not against the whole virus. In this setting, the negative T-cell response in healthy VZV-seropositive subjects should be further investigated using different peptide pools or the whole virus. 

VZV-specific antibody response and T-cell response against VZV antigens measured by ex vivo ELISpot assay were evaluated, and correlation between the two responses was investigated. No correlation was observed between IgG VZV-specific antibody titer and gE or IE63 specific T-cell responses, according to the results obtained by Rondaan et al. [22]. It has been suggested that low antibody titers or B cell immunodeficiency are not related to severe varicella cases [12]. Furthermore, T-cell responses measured by ex vivo, and cultured ELISpot assays did not correlate, further supporting that the two memory T-cell subpopulations act differently. Although ELISpot is considered a sensitive method for studying T cell immunity [23,24], it does not allow phenotypical discrimination of individual cytokine-producing cells. Therefore, we also performed analyses of T cell IFNγ production by flow cytometry. According to the availability of PBMC, VZV-specific T effector memory response to gE and IE63 was investigated in eight VZV-seropositive healthy subjects, observing that T effector memory response is predominantly CD4 mediated rather than CD8 mediated, as previously described [20,21]. 

We observed that T cell responses against gE and IE63 antigens were not statistically different between healthy controls and transplanted patients. Moreover, in some cases, T-cell response against VZV observed in patients at pre-transplant was higher than that observed in healthy subjects. In this setting, increased production of cytokines is a well-known feature of patients with end-stage renal disease, which is thought to be related to the pro-inflammatory state caused by uremia [25,26]. We then monitored VZV-specific T-cell responses during the first six months after transplant, after 60 and 180 days. As expected, a decrease of gE and IE63-specific T-cell response was observed at 60 days, in line with a higher level of immunosuppression [27].

Interestingly, the decrease of immune response between the day of transplant and 60 days after transplant is not statistically significant, while a significant increase has been detected between day 60 and day 180 but not between day 0 and day 180. This significant increase in terms of VZV-specific T-cell responses was observed at day 180 according to the end of the maximum immunosuppression period due to the normalization of the administered therapy leading to a reconstitution of normal immune function.

Even if a reduction of VZV-specific T-cell response was observed after transplant, no episodes of VZV reactivation have been observed in our subsets of transplanted patients. However, four of them (33.3%) developed episodes of Herpes Simplex Virus 1 or Herpes Simplex Virus 2 reactivation in the period of the most intensive immunosuppression. Moreover, it is possible that real cases of VZV reactivation have been underestimated since unreported herpes zoster episodes may be the consequence of an asymptomatic endogenous viral reactivation, as Smetana et al. previously noted to occur in patients receiving renal replacement therapy (RRT) [28]. In this setting, it would be interesting to correlate the role of VZV-specific T-cell response with the risk of VZV reactivation on a larger sample of transplanted patients. This novel ex vivo functional T-cell assay provides a unique opportunity to identify potential herpes zoster-predisposing VZV-specific T-cells in blood specimens of individuals and transplant recipients. Over 90% of adult solid organ transplant (SOT) recipients will be seropositive for VZV. Rates are lower in pediatric transplants [16,17]. Understanding the mechanism underlying the susceptibility may lead to new approaches of herpes zoster prevention in these patients, also for other herpesviruses reactivations.

## 5. Conclusions

The determination of lymphocyte-specific T-cell responses may be a simple method to identify, in healthy subjects and solid transplant patients, the risk of developing opportunistic infections. It may therefore be useful for the stratification of patients according to the risk of reactivation, for the introduction of prophylaxis therapies and for the modulation of immunosuppressive therapy. This immunological monitoring strategy thus represents an important milestone for the personalized medicine perspectives and management of the patient in the post-transplant period.
